# Bispecific NKG2D-CD3 and NKG2D-CD16 Fusion Proteins as Novel Treatment Option in Advanced Soft Tissue Sarcomas

**DOI:** 10.3389/fimmu.2021.653081

**Published:** 2021-04-14

**Authors:** Ilona Hagelstein, Martina S. Lutz, Moritz Schmidt, Jonas S. Heitmann, Elke Malenke, Yanjun Zhou, Kim L. Clar, Hans-Georg Kopp, Gundram Jung, Helmut R. Salih, Melanie Märklin, Clemens Hinterleitner

**Affiliations:** ^1^ Clinical Collaboration Unit Translational Immunology, German Cancer Consortium (DKTK), Department of Internal Medicine, University Hospital Tuebingen, Tuebingen, Germany; ^2^ Cluster of Excellence iFIT (EXC 2180) “Image-Guided and Functionally Instructed Tumor Therapies”, University of Tuebingen, Tübingen, Germany; ^3^ Department of Medical Oncology and Pneumology (Internal Medicine VIII), University Hospital Tuebingen, Tuebingen, Germany; ^4^ Department of Hematology and Oncology, Eberhard Karls University Tuebingen, Children’s Hospital, Tuebingen, Germany; ^5^ Robert Bosch Center for Tumor Diseases (RBCT) Robert Bosch Cancer Center, Stuttgart, Germany; ^6^ Department of Hematology, Oncology, Clinical Immunology and Rheumatology, University Hospital Tuebingen, Tuebingen, Germany; ^7^ Department for Immunology, Eberhard Karls University, Tuebingen, Germany

**Keywords:** sarcoma, NKG2DL, CD3, CD16, fusion protein, mAb, immunotherapy

## Abstract

Soft tissue sarcoma (STS) constitutes a rare group of heterogeneous malignancies. Effective treatment options for most subtypes of STS are still limited. As a result, especially in metastatic disease, prognosis is still dismal. The ligands for the activating immunoreceptor NKG2D (NKG2DL) are commonly expressed in STS, but generally absent in healthy tissues. This provides the rationale for utilization of NKG2DL as targets for immunotherapeutic approaches. We here report on the preclinical characterization of bispecific fusion proteins (BFP) consisting of the extracellular domain of the NKG2D receptor fused to Fab-fragments directed against CD3 (NKG2D-CD3) or CD16 (NKG2D-CD16) for treatment of STS. After characterization of NKG2DL expression patterns on various STS cell lines, we demonstrated that both NKG2D-CD16 and NKG2D-CD3 induce profound T and NK cell reactivity as revealed by analysis of activation, degranulation and secretion of IFNγ as well as granule associated proteins, resulting in potent target cell lysis. In addition, the stimulatory capacity of the constructs to induce T and NK cell activation was analyzed in heavily pretreated STS patients and found to be comparable to healthy donors. Our results emphasize the potential of NKG2D-CD3 and NKG2D-CD16 BFP to target STS even in an advanced disease.

## Introduction

Soft tissue sarcomas (STS) comprise a rare, heterogenic group of malignancies derived from tissues of mesenchymal lineage (1). With more than 100 different entities, biological and clinical characteristics in STS vary from low-grade tumors to highly aggressive cancers with an enormous metastatic potential (1, 2). Reflecting its heterogeneity, genetic alterations in STS are highly variable. Some STS histotypes are characterized by distinct genetic alterations including *EWS-ATF1* in Ewing sarcomas, c*KIT* mutations in GISTs or *PAX3-FKHR* in alveolar rhabdomyosarcomas (3). Complex karyotypes, typically associated with a worse clinical prognosis are frequently related to *PTEN*, *RB1*, *BRCA2*, *PIK3CA* or *APC* mutations (4–6). Interestingly, some STS subtypes have recently been associated with epigenetic dysregulations triggered by a single or small number of genetic alterations (3, 7).

Although therapeutic options have significantly increased over the recent years, long-term survival, especially in a metastatic disease, is still very limited (8, 9). Even if first-line treatment with anthracyclines and alkylating agents shows favorable results regarding progression-free survival in young STS patients, overall survival is only marginally affected (10). Due to the complex and heterogeneous biology of sarcomas even molecular targeted therapy shows only partially success (11).

Immunotherapy of cancer has made sustainable progress in the past few years. While novel immunotherapeutic strategies have already moved into standard clinical practice for various neoplasms, a similar development is lagging behind for the treatment of sarcoma (12). However, since the tumor microenvironment (TME) of sarcomas reportedly is infiltrated by a high amount of different immune cell populations, implementation of immunotherapeutic approaches seems promising (11, 13). Beyond immune-checkpoint inhibitors targeting PD-1 and PD-L1, modified T and NK cell therapies have recently shown first encouraging results in STS and other solid tumors (14).

Bispecific antibodies represent another concept of anti-cancer immune therapy. Their mode of action is to directly attract immune effector cells like NK and T cells to the tumor cell resulting in lysis of the tumor cells (15). In order to ensure treatment efficiency and therapeutic safety of these molecules, it is essential to identify reliable target antigens, broadly expressed on tumor tissue while absent in healthy tissues. Several ligands for the activating immunoreceptor NKG2D (natural killer group 2D) (NKG2DL) including the UL16-binding protein (ULBP) family and MHC class I-related chain (MIC) proteins have been described to be selectively overexpressed in human cancer cells like acute myeloid leukemia, lung cancer, ovarian and breast cancer and sarcomas (15). Moreover, the expression of NKG2DL on tumor cells can be upregulated as a result of chemotherapy and radiotherapy, which induce DNA damage pathways (16). Therefore, this protein family reflects an encouraging target for bispecific antibodies (17, 18).

**Figure 1:**
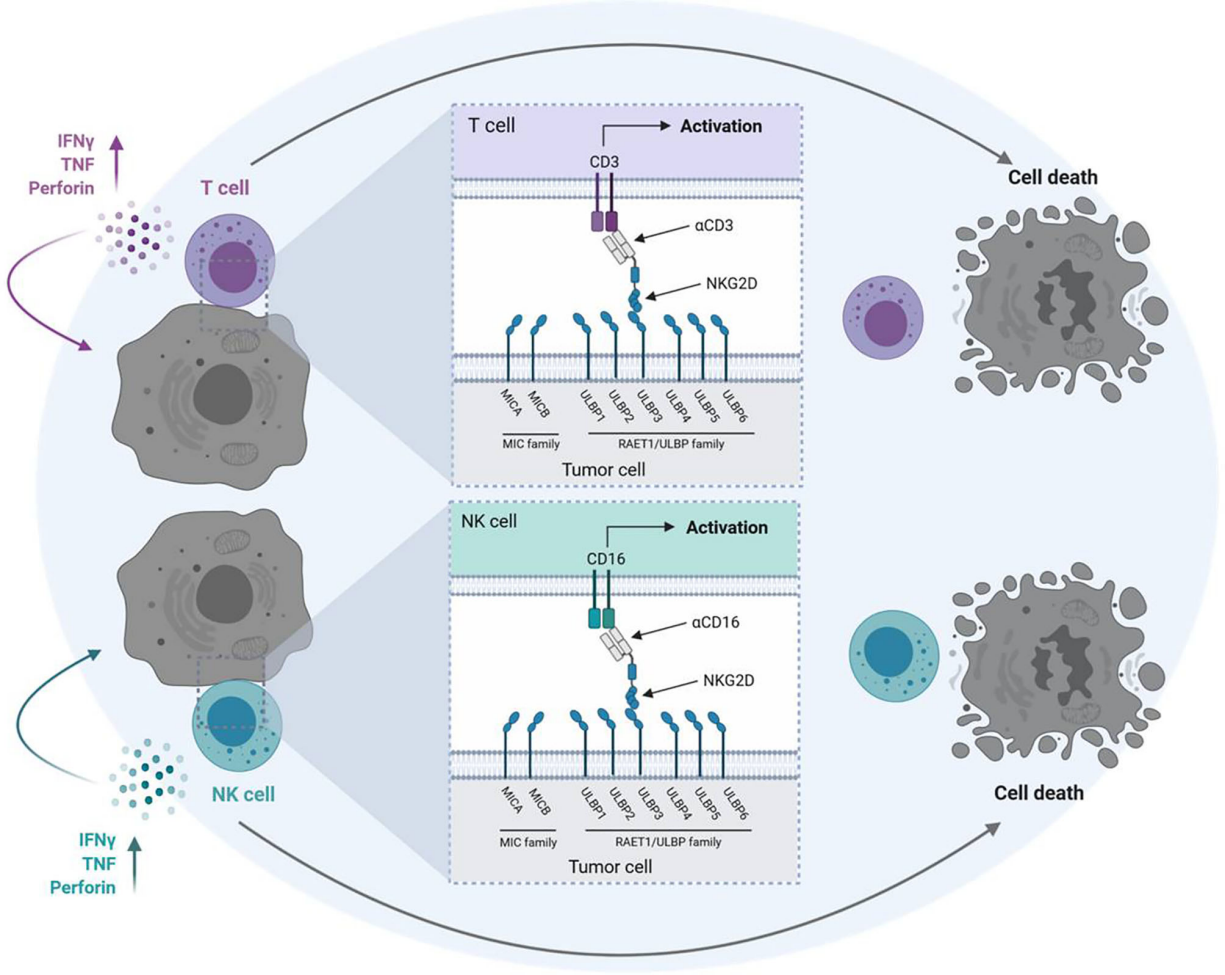
Mechanism of action of NKG2D-CD16/CD3 fusion proteins. Schematic illustration of BFP consisting of the extracellular domain of the NKG2D receptor fused to Fab-fragments directed against CD3 (NKG2D-CD3) or CD16 (NKG2D-CD16). Binding of NKG2D to NKG2DL (MICA/B, ULBP1-6) leads to activation of T cells and NK cells *via* anti-CD3 and anti-CD16 Fab-fragments and subsequent lysis of tumor cells. The graphic was created with BioRender software (BioRender.com, Toronto, Canada).

We previously reported on a Fc-optimized NKG2D-IgG_1_ fusion protein (NKG2D-Fc-ADCC) that allows simultaneous targeting of all NKG2DL. Compared to a construct with a wildtype Fc part our NKG2D-Fc-ADCC showed enhanced antitumor efficacy in acute leukemia and breast cancer (19, 20). However, our NKG2D-Fc-ADCC construct lacks the ability to actively stimulate T cells with their, compared to NK cells, higher effector potential. As a consequence, we developed NKG2D-CD16 and NKG2D-CD3 bispecific fusion proteins (BFP) consisting of the extracellular domain of NKG2D fused to anti-CD16 or anti-CD3 Fab-fragments instead of an optimized Fc part, which allow for recruitment of NK cells and T cells, respectively (**Figure 1**). NKG2D-CD16 and NKG2D-CD3 BFP were previously shown to induce potent lysis of acute myeloid leukemia cells (21). Considering the reported expression of NKG2DL in STS (22, 23), our NKG2D-CD16 and NKG2D‑CD3 BFPs might offer a new promising approach in sarcoma treatment (11, 18).

In the present study we provide preclinical evidence that an effective treatment with NKG2D-CD16 and NKG2D-CD3 BFPs might be possible even in heavily pretreated STS patients.

## Material and Methods

### Production and Purification of NKG2D Fusion Proteins

The NKG2D-CD16 and NKG2D-CD3 constructs were generated as described previously (21). In brief, the constructs were produced in SP2/0-Ag14 cells (American Type Culture Collection, Manassas, VA) and purified from culture supernatant by HiTrap KappaSelect affinity chromatography (GE Healthcare, Munich, Germany) followed by preparative size exclusion chromatography on Superdex HiLoad. Analytical size exclusion chromatography (Superdex 200R PC3.2/30, GE Healthcare) and 4-12% gradient SDS-PAGE (Invitrogen, Carlsbad, CA) was performed to confirm the quality and exclude aggregation of purified bispecific fusion proteins. Endotoxin levels were measured with EndoZyme II (BioMerieux, Marcy-l’Étoile, France) according to the manufacturer’s instructions and below endotoxin levels EU=0.1.

### STS Cell Lines

Human sarcoma cell lines SW1353, SaOs, SW872, RD-ES and SW982 were obtained from ATCC (American Type Culture Collection). Previously described genetic alterations are given in (**Table S1**). Cells were tested routinely for mycoplasma contamination every three months. Authenticity was determined by validating the respective immunophenotype described by the provider using flow cytometry.

### Primary Patient Material

To obtain patient-derived sarcoma cells (**Table S2**), an outgrowth culture from resected primary tumors was generated. Tumors were cut in small fragments of 1-2 mm^3^ and washed with PBS (LifeTechnologies, Carlsbad, CA). All sarcoma samples were cultured in Dulbecco’s minimal essential medium (DMEM, LifeTechnologies) containing 4.5 g/l glucose (Thermo Fisher Scientific, Waltham, MA) or Eagle’s Minimum Essential Medium (EMEM, LifeTechnologies) supplemented with 10% fetal bovine serum (Biochrom, Berlin, Germany) and 1x antibiotic-antimycotic solution (LifeTechnologies) with 100 units/ml penicillin, 100 µg/ml streptomycin and 0.25 µg/ml amphothericin B (Thermo Fisher Scientific). After reaching optimal density, cells were cryopreserved with 90% FBS and 10% DMSO (Merck, Darmstadt, Germany) in liquid nitrogen.

Blood samples from 16 consecutive sarcoma patients treated at the Department of Medical Oncology and Pneumology (10-11/2020) were included in our study. The patient characteristics in detail are given in **Table 1** and the respective treatment regimens are given in **Table S3**. Peripheral Blood Mononuclear cells (PBMC) of healthy donors or sarcoma patients were isolated by density gradient centrifugation (Biocoll; Biochrom) and viably stored in liquid nitrogen. After thawing, PBMC were cultured for 18-24 h in media prior the use in functional experiments.

**Table 1:** Patient characteristics.

Clinical characteristics	Total
	(n=16)
**Gender**	
Female sex, n (%)	5 (31)
**Age**	
Age in years, mean–yr. ± SD (range)	49,5 ± 18.5 (19-72)
**TNM classification, n (%)**	
Stage	
T0	0
T1	3 (18)
T2	8 (50)
T3	2 (13)
T4	2 (13)
Tx	1 (6)
Node	
N0	8 (50)
N1	3 (18)
N2	0
N3	1 (6)
Nx	4 (25)
Metastasis	
M0	10 (63)
M1	6 (37)
**Histological subtype**	
Ewing sarcoma	2 (13)
Osteosarcoma	6 (37)
Chondrosarcoma	1 (6)
SynovialSarcoma	1 (6)
Liposarcoma	2 (13)
Leiomyosarcoma	2 (13)
Soft tissue sarcoma not otherwise specified	1 (6)
Angiosarcoma	1 (6)
	
**Histological grading, n (%)**	
G1	0
G2	5 (31)
G3	5 (31)
Unknown	6 (37)
	
**Treatment, n (%)**	
Anthracycline	6 (37)
Anthracycline/platinum	5 (31)
Vincaalcaloid/anthracycline/ topoisomerase II inhibitor	2 (13)
Vincaalcaloid/actinomycin	1 (6)
Alcaloid	1 (6)
Purine analogue	1 (6)
	
**Peripheral blood count**	
Leucoytes (1/µl)	7465 ± 2933
Hb (g/dL)	9.07 ± 1.46
Thrombocytes (10^3^/µl)	239.8 ± 117.9
	
**Therapy line at the time-point of blood sampling**	
Neoadjuvant	4 (25)
Adjuvant, n (%)	12 (75)
1st line, n (%)	13 (81)
2nd line, n (%)	1 (6)
> 2nd line, n (%)	2 (13)
	
**Therapy line at the time-point of blood sampling**	
Neoadjuvant	4 (25)
Adjuvant, n (%)	12 (75)
	

TNM, tumor (T), nodes (N), metastases (M); Hb, hemoglobin.

Written informed consent, in accordance with the Helsinki protocol, was given in all cases.

### PCR

Total RNA of 1-3 million sarcoma cells was isolated using the High Pure RNA Isolation Kit (Roche, Basel, Switzerland) and cDNA synthesis was done using FastGeneScriptase II (NIPPON Genetics Europe, Düren, Germany) according to the manufacturer’s instructions. Primer sequences for MICA, MICB, ULBP1-4 and GAPDH were used as previously described (24, 25) (**Table S4**). Reverse transcriptase–polymerase chain reaction (RT-PCR) was performed as described previously (26). Quantitative PCR (q-PCR) was performed using PerfeCTa SYBR Green FastMix (Quanta Biosciences Beverly, MA) with a LightCycler 480 (Roche) instrument.

### Immunohistochemistry and Immunofluorescence

For H&E staining sarcoma tissue samples were paraffin-embedded. 4 µm paraffin-embedded samples were stained with hematoxylin and eosin following standard protocols. For immunofluorescence staining, SW1353 and RD-ES cells were incubated with PBMC of healthy donors (ratio 2.5:1). After incubation, media was aspirated and cells were fixed using 4% paraformaldehyde (PFA) in PBS (10 min at 20°C). After PFA was aspirated, cells were washed three times using PBS + 0.1% Tween20 (PBST). Cells were blocked using a Bovine Serum Albumin (BSA) blocking solution containing 5% BSA, 0.2% Triton X-100 and 0.1% Tween20 for 60 min. Blocking buffer was aspirated and cells were washed three times with PBST. Staining was conducted using a rabbit α-Tubulin antibody (11H10, 1:500, Cell Signaling, Danvers, MA) and murine mAbs against CD3 (clone OKT3, 1:25, Biolegend, San Diego, CA), CD16 (clone #1001049, 1:25, R&D systems, Minneapolis, MN) or Perforin (clone δg9, 1:250, BD Pharmingen, Heidelberg, Germany) (overnight at 4°C), followed by a Alexa-Fluor 488 labeled anti-mouse (1:500) and Alexa-Fluor 594 labeled anti-rabbit (1:500) antibody (both Invitrogen). For staining of actin, fluorescein conjugated Phalloidin (1:1000, Abcam, Cambridge, Great Britain) was used according to manufacturer’s instructions. Slides were mounted in fluorescent mounting medium; DAPI was used for counter-staining. Pictures were acquired using an Olympus BX63 microscope and a DP80 camera (Olympus, Shinjuku, Japan).

### Flow Cytometry

For studies on NKG2DL surface expression and NKG2D binding, cells were stained with respective unconjugated mAbs (10 µg/mL) for single NKG2DL or the corresponding isotype controls as described previously (19) and biotinylated NKG2D-Fc (20µg/mL) or the corresponding isotype control as described previously (24) followed by a goat anti-mouse PE conjugate (Dako, Glostrup, Denmark) or a streptavidin-conjugated PE conjugate (LifeTechnologies, Carlsbad, CA), respectively.

PBMC subsets of sarcoma patients and healthy control donors were identified by counterstaining with CD3-APC/Fire750, CD4-Pacific Blue, CD8-BV605, CD14-BV785, CD16-APC, CD19-FITC, CD56-PeCy7 and HLA-DR-BV650 (BioLegend).

For studies on NK cell activation and degranulation, the fluorescently labeled mAbs CD69-PE and CD107a-PE (BD Pharmingen) as well as CD3-APC/Fire750, CD4-APC, CD8-FITC and CD56-PeCy7 or CD56-BV711 (BioLegend) were used. For Flow cytometric analysis of target cell lysis, sarcoma cells were loaded with 2.5 µM CellTrace™ Violet cell proliferation dye (Thermo Fisher Scientific, Waltham, MA) and cultured with PBMC (E:T 2.5:1) in the presence or absence of the fusion proteins (2.5 µg/mL). Standard calibration beads (Sigma-Aldrich, St. Louis, MO) were used to ensure the analysis of equal assay volumes and therefore to account for the number of target cells that had vanished from the culture.

Dead cells were excluded from analysis by 7-AAD (BioLegend). Measurements were performed using a FACS Canto II or FACS Fortessa (BD Biosciences, San Diego, CA) and data analyzed using the software FlowJo V10 (FlowJo LCC, Ashland, OR).

### Analysis of NK and T Cell Activation and Degranulation

To determine the activation status of PBMC obtained from sarcoma patients and healthy donors in the absence of target cells, 7.5 μg/mL anti-NKG2D mAb (6H7) was coated on 96-well plates overnight and, after rinsing with PBS, followed by 2 h incubation with or without the NKG2D fusion proteins (10 μg/mL each). Subsequently, mAb solution was removed and 5×10^5^ PBMC were added, incubated for 24 h and analyzed by flow cytometry for CD69 expression.

To determine activation and degranulation in the presence of target cells, 200.000 sarcoma cells were cultured with allogenic PBMC of healthy donors (E:T ratio 2.5:1) for 4 h and 24 h, respectively. For analysis of degranulation after 4 h, CD107a-PE (1:25), BD GolgiStop and BD GolgiPlug (1:1000, both BD Biosciences) were added to the coculture of PBMC and sarcoma cells. Analysis was conducted using flow cytometry.

### Analysis of Cytokine Expression and Secretion

PBMC of healthy donors were cultured with sarcoma cells (E:T ratio 2.5:1) in the presence or absence of treatment (2.5 µg/mL). After 4 h, supernatants were harvested and secretion of IFNγ, Granzyme A, Perforin and Granulysin was then analyzed using Legendplex assays (BioLegend). For analysis of cytokine expression, PBMC were treated as described above and cultured for 24 h. Monensin (GolgiStop, BD Biosciences) was added 12 h prior to flow cytometric analysis. Cells were stained using the Cytofix/Cytoperm Fixation/Permeabilization Solution Kit (BD Biosciences). For detection of intracellular levels of IFNγ (clone b27) and Perforin (clone dG9), fluorescence-conjugated antibodies (both from BioLegend) were used in 1:20 dilutions.

### Cytotoxicity Assays

Lysis of sarcoma cells by PBMC of healthy donors in the presence or absence of the fusion proteins (2.5 µg/mL) was assessed by 2 hour Europium based cytotoxicity assays as previously described (20). In brief, sarcoma cells were labeled with DELFIA^®^ BATDA for 1 h (Perkin Elmer, Waltham, MA). After labeling, the cells were incubated with PBMC at the indicated E:T ratios. After incubation, 20 µl supernatant of each sample was mixed with 200 µl DELFIA^®^ Europium solution (PerkinElmer). Subsequent, samples were measured using a VICTOR (Wallac Oy, Finland)

Specific lysis was calculated as follows:

100 × (experimental release − spontaneous release)/(maximum release − spontaneous release).

Long-term cytotoxicity analyses were performed using the IncuCyte^®^ S3 Live-Cell Analysis System (Essenbioscience, Sartorius, Göttingen). Sarcoma cells were cultured with PBMC of healthy donors (E:T ratio 5:1) with or without the indicated treatments (2.5 µg/mL each). Live cell imaging pictures were taken every 4 h with 10x magnification. To quantify living cells, confluences were normalized to the respective measurement at T=0 h.

### Statistics

If not indicated otherwise, values depict means ± standard deviation (SD). For continuous variables student’s t test, Mann-Whitney U test or one-way ANOVA was used. For statistical analysis, GraphPad Prism 8 (GraphPad Software, San Diego, CA) was used. All statistical tests were considered significant when *p* was below 0.05.

## Results

### Characterization of NKG2DL Expression in Sarcoma Cells

**Figure 2:**
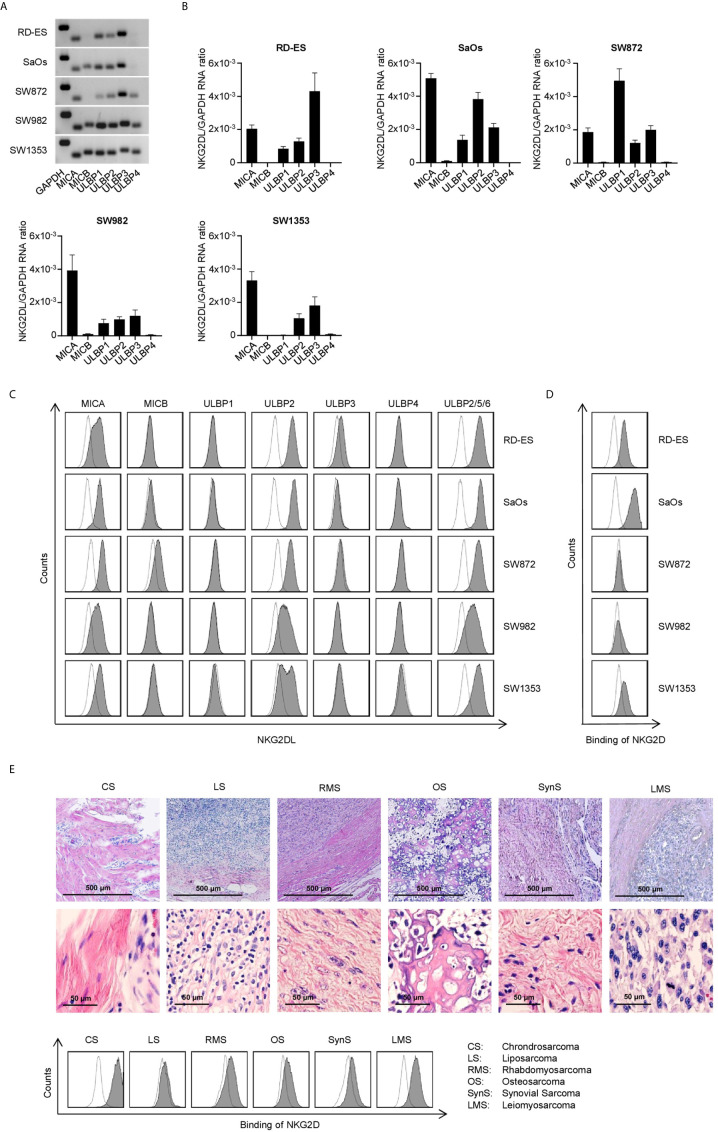
Characterization of NKG2DL expression in STS cell lines. **(A)** MICA, MICB and ULBP1-4 mRNA expression was determined *via* RT-PCR with GAPDH serving as control. PCR products were visualized by agarose gel electrophoresis. **(B)** Relative mRNA expression of MICA, MICB and ULBP1-4 in five different STS cell lines was determined as described in the method section. Results for n=3 experiments are shown. **(C)** Surface expression of MICA, MICB, ULBP1-4 and ULBP2/5/6 on the indicated cell lines was analyzed by flow cytometry. mAb against the depicted NKG2DL are shown as shaded peaks, corresponding isotype controls are shown as open peaks. **(D)** Binding of NKG2D to the surface of sarcoma cells lines was analyzed by flow cytometry using an NKG2D-Fc-chimera (shaded peaks) and the corresponding isotype control (open peaks). **(E)** H&E staining of paraffin-embedded tissue sections from primary sarcoma tissue was performed (upper panel). Patient-derived sarcoma cells dissociated from the primary tumor were analyzed by flow cytometry using a biotinylated NKG2D-Fc-chimera (shaded peaks) and the corresponding isotype control (open peaks) followed by strep PE.

Since different subgroups of STS have been reported to express varying patterns of NKG2DL (22, 23), we initially characterized the NKG2DL mRNA expression in rhabdomyosarcoma (RD-ES), osteosarcoma (SaOs), liposarcoma (SW872), synovial sarcoma (SW982) and chondrosarcoma (SW1353) cell lines. This mRNA analysis confirmed expression of at least one NKG2DL with varying expression patterns in all tested STS cell lines (**Figures 2A, B** **)**. Whereas MICA, ULBP2 and 3 mRNA levels were found to be broadly expressed in all STS cell lines, MICB and ULBP4 mRNA signatures were only detected to a very low amount.

Next we determined the surface protein expression using specific mAbs against MICA, MICB, and ULBP1-4. For analysis of the expression of ULBP5 and 6, an Ab recognizing both in combination with ULBP2 was used. The detectable levels on the surface of the cell lines did not directly mirror the mRNA expression levels detected upon PCR analysis, which is in line with the fact that NKG2DL expression underlies posttranslational regulation (27, 28). MICA and ULBP2 showed the highest prevalence as well as surface expression level among the tested cell lines, whereas ULBP1 and ULBP4 could not be detected (**Figure 2C**). Determination of NKG2DL surface levels using a NKG2D-Fc chimera for simultaneous analysis of all different NKG2DL showed different staining intensities between the cell lines (**Figure 2D**). Each single NKG2DL is characterized by a specific affinity for the NKG2D receptor (29), which results in varying intensities of mAB-based staining compared to the NKG2D-Fc fusion protein staining. Based on the results for NKG2DL expression, we chose the cell lines SaOs, RD-ES and SW1353 for further functional evaluation. In addition, we analyzed binding of NKG2D on 6 patient-derived sarcoma cells from 6 STS subgroups (**Table S2**). Patient-derived sarcoma cells were analyzed by flow cytometry after dissociation and an outgrowth culture from resected primary tumors. For identification of the tumor tissue sections, H&E stainings of paraffin-embedded tissue was performed additionally. The analysis revealed expression of NKG2DL to different extends with the NKG2D-Fc fusion protein and a mAb NKG2DL cocktail staining (**Figures 2E**, **S1**).

### Modulation of NK and T Cell Binding and Reactivity Against STS cells by NKG2D BFP

**Figure 3:**
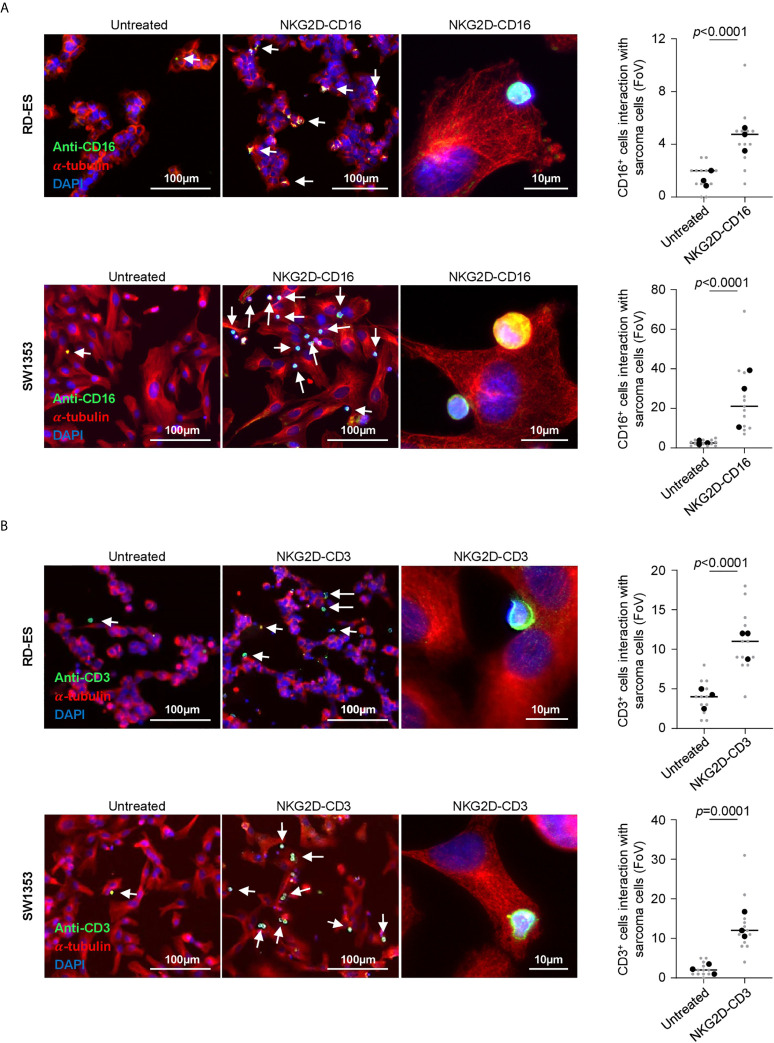
Recruitment of NK and T cells to tumor cells *via* binding of NKG2D-CD3/CD16. The Indicated sarcoma cell lines were cultivated with PBMC of healthy donors (E:T 2.5:1) in the presence or absence of NKG2D-CD3/CD16 (2.5 µg/ml) and subsequently stained for α-Tubulin and **(A)** CD16 or **(B)** CD3. DAPI was used for counterstaining. Interaction of CD16^+^ and CD3^+^ cells was quantified by counting triple positive cells located at sarcoma cells. Grey and black dots represent individual analysis per FoV (n=12) out of three independent experiments.

Next we analyzed the efficacy of NKG2D-CD16 and NKG2D-CD3 to mediate T and NK cell recruitment to the sarcoma cells. PBMC of healthy donors were incubated with RD-ES and SW1353 cells in the presence or absence of NKG2D-CD16 and NKG2D-CD3 BFP. Immune cell recruitment to STS was visually quantified *via* determination of co-localized effector and target cells per field of view (FoV). We observed a significant increase of CD16^+^ cells interacting with sarcoma cells when treated with NKG2D-CD16 in three independent experiments and two different cell lines (RD-ES: *p*<0.0001, SW1353: *p*<0.0001, **Figures 3A**, **S2A**). A similar effect was observed for CD3^+^ cells when RD-ES and SW1353 cells were co-incubated with PBMC and NKG2D-CD3 (RD-ES: *p*<0.0001, SW1353: *p*<0.0001, **Figures 3B**, **S2B**), confirming enhanced recruitment of NK and T cells upon treatment with the respective BFP.

**Figure 4:**
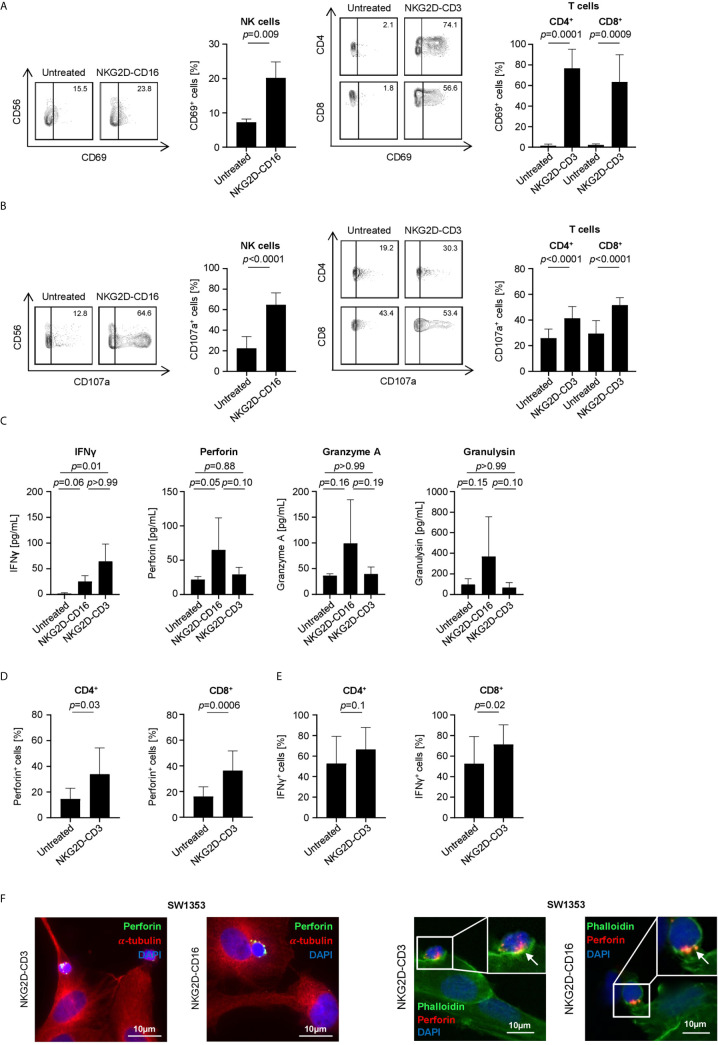
Induction of NK and T cell reactivity by NKG2D-CD16/CD3 against sarcoma cells. PBMC of healthy donors were cultured with or without sarcoma cells at an E:T ratio of 2.5:1 in the presence or absence of NKG2D-CD16/CD3 (2.5 µg/mL). **(A)** Activation of NK cells and CD4^+^ and CD8^+^ T cells was determined by expression of CD69 after 24 h. In the left panels exemplary flow cytometry results obtained with SaOs and in the right panel combined data with sarcoma cell lines SaOs, RD-ES and SW1353 and with PBMC of 4 different donors are shown. **(B)** Degranulation of NK cells and CD4^+^ and CD8^+^ T cells was determined by expression of CD107a after 4 h. In the left panels exemplary flow cytometry results obtained with SaOs and in the right panel combined data with sarcoma cell lines SaOs, RD-ES and SW1353 and with PBMC of four independent donors are shown. **(C)** Supernatants were analyzed for IFNγ, Granzyme A, Perforin and Granulysin after 4 h by Legendplex assays. Shown are pooled results with sarcoma cell lines SaOs, RD-ES and SW1353 and with PBMC of two independent donors. **(D, E)** Intracellular expression of Perforin **(D)** and IFNγ **(E)** was analyzed after 24 h by flow cytometry. **(F)** Immunofluorescent staining was performed after 1 h (for NKG2D-CD16 treatment) and 3 h (for NKG2D-CD3 treatment). In the left panels, cells were stained for α-Tubulin (red) and the granular marker Perforin (green). In the right panels, cells were stained for actin with Phalloidin (green) and perforin (red).

Next we characterized the potential of our constructs to induce effector cell reactivity against sarcoma cells. To this end, PBMC of healthy donors were cultured with SW1353, RD-ES or SaOs cells in the presence or absence of BFP. Flow cytometry analysis of CD69 on NK and T cells revealed that both constructs significantly induced activation upon treatment with NKG2D-CD16 and NKG2D-CD3, respectively (NK cells: *p*=0.009, CD4: *p*=0.0001, CD8: *p*=0.0009, **Figure 4A**). In line, determination of CD107a upregulation confirmed that NKG2D-CD16 and NKG2D-CD3 potently induced degranulation of NK and T cells, respectively (NK cells: *p*<0.0001, CD4: *p*<0.0001, CD8: *p*<0.0001, **Figure 4B**). Additional analysis of supernatants by Legendplex assays showed a significant increase in IFNγ secretion after treatment with NKG2D-CD3, while secretion of Perforin was profoundly increased in supernatants of NKG2D-CD16 treated cells. Furthermore there was a tendency to increased Granzyme A and Granulysin secretion after NKG2D-CD16 treatment (**Figure 4C**). To determine induction of Perforin and IFNγ by treatment with the BFP, we analyzed the effector cells using intracellular flow cytometry. Expression of Perforin was significantly increased in both CD4^+^ and CD8^+^ T cells (**Figure 4D**). IFNγ expression was potently induced in CD8^+^ T cells with only marginal effects for CD4^+^ T cells (**Figure 4E**). NK cells constitutively contained large amounts of Perforin and IFNγ (**Figure S3**). In addition to Perforin detection *via* Legendplex assay and intracellular flow cytometric staining, we performed immunofluorescence staining of Perforin in CD16^+^ and CD3^+^ effector cells. Interestingly, a bright staining for Perforin could be detected after 1 h and 3 h of treatment with NKG2D-CD16 and NKG2D-CD3, respectively (**Figures 4F**, **S4** **).**

### Subpopulations of PBMC and Modulation of NK and T Cell Reactivity in Advanced STS Patients

**Figure 5:**
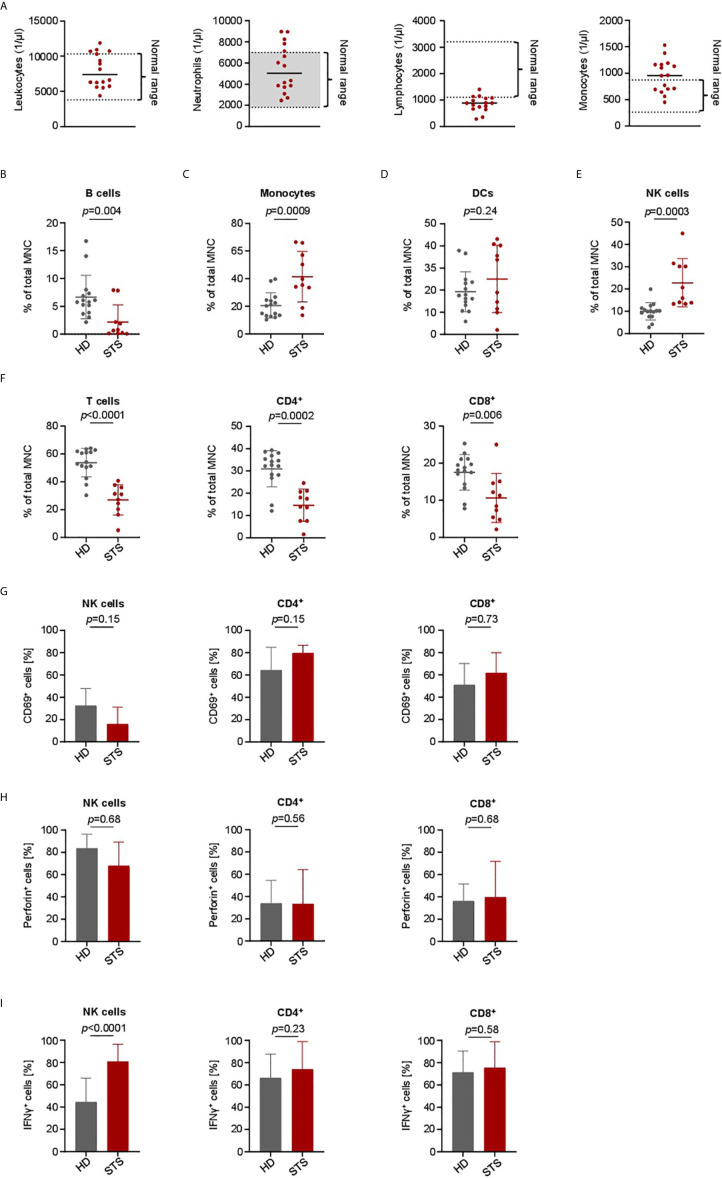
Immune cell characterization and lymphocyte activation capacity in advanced STS patients and healthy donors. PBMC were collected from healthy donors (HD) and patients with advanced STS (STS). **(A)** Immune cell counts for leukocytes, neutrophils, lymphocytes and monocytes at time point of PBMC collection is shown (n=16). **(B–F)** Indicated cell types were identified by counterstaining of PBMC from HD (n=13) and from STS patients (n=10) for CD3, CD4, CD8, CD14, CD16, CD19, CD56 and HLA-DR and subsequently analyzed by flow cytometry and displayed as percentage of mononuclear cells (MNC). **(G)** To analyze the effector capacity of STS patient effector cells, bispecific NKG2D-CD16/CD3 fusion proteins were immobilized to plastic as described in the methods section and incubated with PBMC of healthy donors (n=5) or sarcoma patients (n=4). Expression of CD69 as marker for activation was determined after 24 h using flow cytometry. Percentage of CD69 positive NK cells after treatment with NKG2D-16 and percentage of CD69 positive CD4^+^ and CD8^+^ T cells after treatment with NKG2D-CD3 are shown. **(H, I)** PBMC from HD (n=6) and STS patients (n=5) were cultured with sarcoma cells (SaOs, RD-ES and SW1353) and treated with NKG2D-CD16/CD3 (2.5 µg/ml) for 24 h. Intracellular Perforin (h) and IFNγ **(I)** expression was analyzed by flow cytometry for CD4^+^ and CD8^+^ T cells and NK cells.

To determine whether there are disease and treatment-associated alterations in the immune cell subsets of sarcoma patients that could impair treatment efficacy of our BFP, we comparatively analyzed PBMC of healthy donors (HD) and sarcoma patients (STS). Of note, during sample generation all tested STS patients were treated with intense neo- or adjuvant cytotoxic chemotherapy. Specific details on treatment regimens of all STS patients are given in **Table 1** and **Table S3**. In our pretreated STS cohort we observed normal counts for leukocytes, neutrophils and monocytes (**Figure 5A**). Due to the chronic treatment-related toxicity we detected slightly decreased total numbers of lymphocytes. To further examine distribution of the main PBMC subsets including B cells (CD19^+^), Monocytes (CD14^+^), Dendritic cells (DCs) (HLA-DR^+^), NK cells (CD3^-^CD56^+^) and T cells (CD3^+^CD4^+^ and CD3^+^CD8^+^), we performed flow cytometric analyses. Compared to healthy donors, we observed a treatment-related decrease of B and T cells (**Figures 5B, F** **)**. In case of T cells, the CD8^+^ subset was less affected than the CD4^+^ subset (**Figure 5F**). The proportion of DCs was similar in the two cohorts (**Figure 5D**). In contrast, increased levels of Monocytes and NK cells were observed in the STS cohort (**Figures 5C, E** **)**. A more detailed analysis of NK cell subsets revealed that in particular the proportion of the CD56^dim^CD16^+^ subset was increased (**Figure S5**).

To determine whether NKG2D-CD3 and NKG2D-CD16 could stimulate effector cells of pretreated STS patients in a similar manner as in healthy donors, we employed an experimental setting independent of target cell binding. To this end, PBMC of 5 healthy donors and 4 STS patients were cultured on the immobilized BFP for 24 h and analyzed for CD69 expression by flow cytometry. Although NK cells of STS patients showed slightly decreased CD69 expression after treatment with NKG2D-CD16 compared to healthy donors (**Figure 5F**), this trend did not reach statistical significance (*p*=0.15). Upon treatment with NKG2D-CD3, T cells of STS patients showed slightly higher CD69 expression levels than healthy donors (**Figure 5G**).

Further, the cytotoxic capacity of PBMC from STS patients was analyzed by co-culturing of PBMC from STS patients and healthy donors with sarcoma cells (RD-ES, SaOs, and SW1353) in the presence of NKG2D‑CD3/CD16 for 24 h followed by intracellular staining for Perforin and IFNγ. No difference between healthy donors and STS patients NK and T cells with regard to Perforin expression was observed (**Figure 5H**). In contrast, STS patients exhibit significantly increased IFNγ positive NK cells after treatment with BFP, while comparable IFNγ induction was observed for T cells (**Figure 5I**).

### Induction of Target Cell Lysis by NKG2D Fusion Proteins

**Figure 6:**
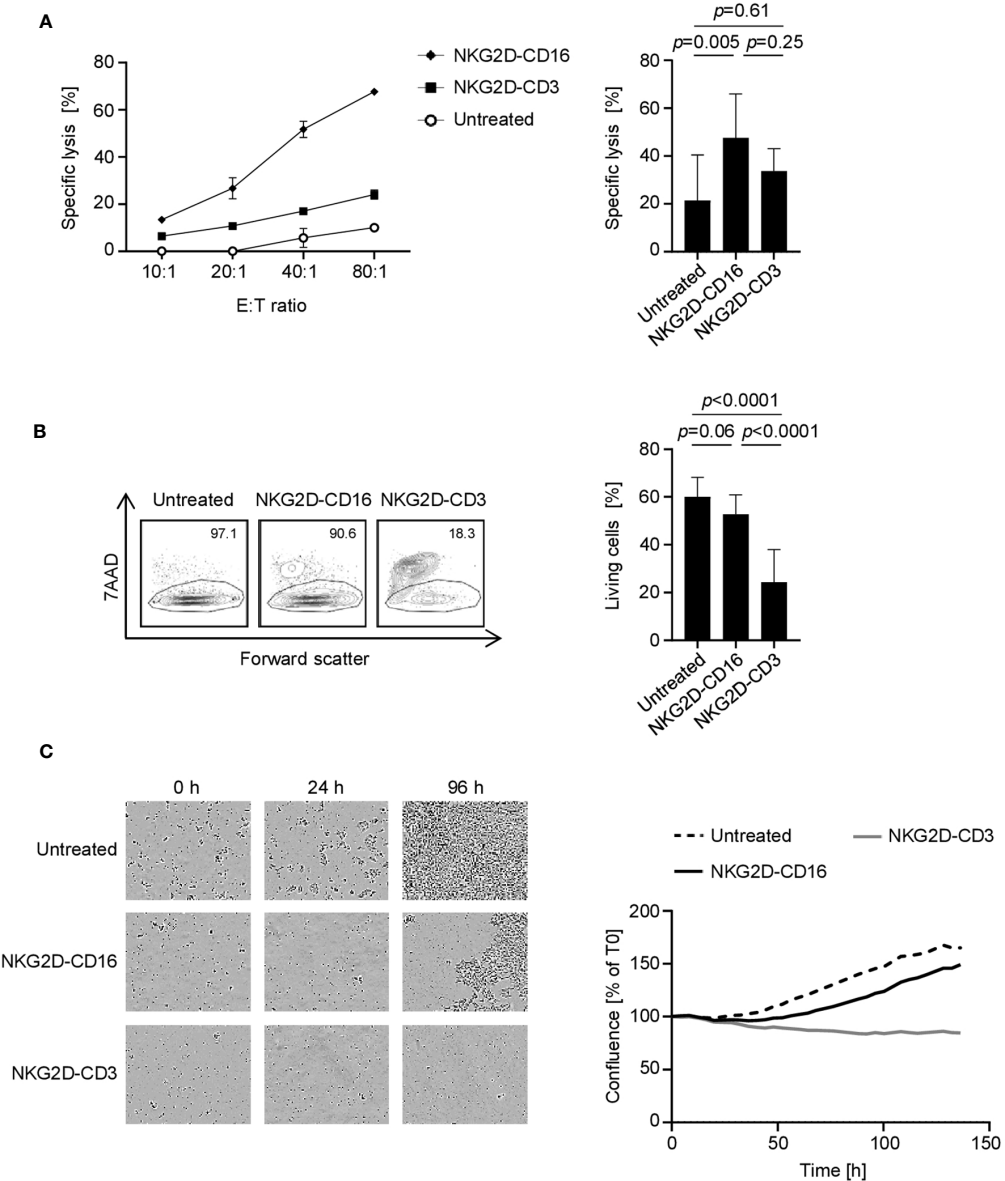
Induction of target cell lysis by NKG2D-CD16/NKG2D-CD3. PBMC of healthy donors (n=4) were incubated with different sarcoma cell lines and treated with the indicated constructs (2.5 µg/mL). **(A)** Lysis of sarcoma cell lines SaOs, RD-ES and SW1353 (n=3) was analyzed by 2 h Europium cytotoxicity assays. On the left, exemplary data obtained with SaOS with different E:T ratios and on the right pooled data obtained with PBMC of healthy donors at an E:T ratio of 40:1 are shown. **(B)** Lysis of sarcoma cell lines SaOs, RD-ES and SW1353 (n=3) was determined by flow cytometry based lysis assay (E:T 2.5:1) using PBMC of healthy donors. In the left panel, exemplary dot plots with SW1353 and one PBMC donor are shown; the right panel depicts pooled data. **(C)** Cell death of sarcoma cells was determined using a live cell imaging system. Cells were incubated with PBMC of healthy donors at an E:T ratio of 5:1 for 136 h. In the left panel, representative pictures at 0, 24, and 96 h are displayed. In the right panel, pooled data with two different cell lines are shown.

Finally, we analyzed whether effector cell activation was mirrored by a lysis of tumor targets. As NKG2D-CD3 and NKG2D-CD16 stimulate T cells and NK cells which differ with regard to efficacy of effector function over time, we performed cytotoxicity assays after various incubation times using different suitable experimental systems. Europium based short-term cytotoxicity assays showed a potent target cell lysis by treatment with NKG2D-CD16 after 2 h, whereas no significant effect was observed with NKG2D-CD3 (**Figures 6A**, **S6A**). After 72 h, flow cytometry based lysis assays **(** **Figures 6B**, **S6B**) revealed profoundly stronger effects with NKG2D-CD3. The finding that the lysis capacity of NKG2D-CD3 occurs at later time points and then surpasses the effects of NKG2D-CD16 was also confirmed by live cell imaging over an incubation period of 136 h (**Figure 6C**).

## Discussion

Although therapeutic options in solid tumors have increased over the recent years, treatment of STS remains still challenging. The complex tumor biology and clinical heterogeneity in STS further complicates the establishment of standardized, efficient treatment concepts (1, 8). In advanced, unresectable or metastatic disease, multimodal high-dose chemotherapeutic agents are commonly used, however with only limited success (10). Accordingly, new therapeutic concepts are urgently needed.

The NKG2D receptor was initially described on human NK and T cells as mediating immune cell activation (30, 31). The ligands of NKG2D include MICA, MICB and ULBP1-6 (32, 33) and are upregulated in multiple tumor entities like acute myeloid leukemia, ovarian-, breast-, lung-cancer, melanoma, glioma and STS (17). Since NKG2DL show a high potential to stimulate immune cells *via* NKG2D and are mainly absent in healthy tissues, multiple treatment approaches modulating the NKG2D system in cancer have been investigated so far (17). Interestingly, expression of several NKG2DL like ULBP1-4 has been identified as negative prognostic marker in STS (18). Recent data have already investigated the potential of NKG2D expressing NK cells to mediate anti-sarcoma responses. However, NKG2D expression was found to be low in both, peripheral and tumor-infiltrating lymphocytes (TIL) like NK cells (18). As a result, genetically modified NK-92 cells over-expressing NKG2D were utilized and showed profound cytotoxicity (18). To overcome the elaborative work required for NK cell generation, in our study we used a novel NKG2D-CD16 BFP to target STS cells. Remarkably, we observed a highly effective tumor cell lysis using NKG2D-CD16 BFP. Since NK cell subpopulations are commonly detected in the TME of STS patients, our preclinical therapeutic approach appears particularly promising (11). Even more important, NKG2D-CD16 overcomes several drawbacks of mAbs with conventional Fc-parts like binding to inhibitory FcγR, which may decrease immunostimulatory potential, or FcγR expressed on non-cytotoxic cells (e.g., platelets and B cells) and FcγR that do not trigger cytotoxicity (e.g. CD16b on granulocytes). Furthermore, NK cell activation is not dependent on the NKG2D receptor which is advantageous, as NKG2D on NK cells can be downregulated in case of sustained activation *via* NKG2DL from tumor cells or other external inhibitory signals (33, 34).

Besides NK cells, cytotoxic T cells play an important role in tumor immunosurveillance (35, 36). Accordingly, we additionally investigated NKG2D-CD3 BFP in STS. Similar to NKG2D-CD16, our NKG2D-CD3 construct showed a profound anti-sarcoma activity as revealed by T cell activation, degranulation, IFNγ and Perforin secretion as well as target cell lysis. Our NKG2D-CD3 construct showed similar enhanced activation of CD4^+^ and CD8^+^ T cells. Since CD4^+^ T cells play an important role in tumor immunity *via* promoting CD8^+^ T cell expansion, generating CD8^+^ T cell memory and priming (37–39) the dual activation of CD4^+^ and CD8^+^ T cells might support anti-tumor efficiency.

When comparing the therapeutic potential of NKG2D-CD16 and NKG2D-CD3 in STS, it is important to note that NKG2D-CD16 showed most potent cytotoxicity after short time points. In contrast, NKG2D-CD3 required longer times to reveal maximum anti-tumor activity. Killing efficiency was more pronounced in T cells stimulated by NKG2D‑CD3 compared to NK cells stimulated *via* NKG2D-CD16. This might reflect the finding that NKG2D-CD3 can induce T cell proliferation. NK cell proliferation however, was not observed after activation *via* NKG2D-CD16 (21).

It should be noted that treatment efficiency of our therapeutic approach might depend on the expression pattern of NKG2DL on tumor cells. This is reflected by the different binding capacity of NKG2D in our tested STS cell lines. The binding affinity of NKG2D to its repertoire of ligands varies from 10^-6^ to 10^‑9^ mol/L (40). Since some NKG2DL only share approximately 25% amino acid homology, this might reflect an “adaptive fit” mechanism (40). In addition, allelic variants of some NKG2DL like MICA show large differences in NKG2D binding, which may affect their efficacy in triggering NK and T cell reactivity (33). Furthermore it has been reported that glycosylation of MICA enhanced complex formation with NKG2D (33). This might reflect our finding that STS cell lines used in this study showed different binding of NKG2D. Nevertheless, treatment strategies which target NKG2DL on tumor cells are promising, as revealed by results obtained e.g. by targeting NKG2D-L with chimeric antigen receptor T (CAR-T) cells (NKR-2) (NCT03018405), (CYAD-02) (NCT04167696). Other promising constructs which utilize the extracellular domain of NKG2D to target NKG2DL expressed on various tumors are NKG2DL-targeted Bispecific T-cell Engagers (NKG2D-BiTE) (41–43).

On a clinical background, one might speculate that lower NK cell activation *via* NKG2D-CD16 correlates with lower rates of cytokine-release dependent side effects. As a result, NKG2D-CD16 might be promising in older STS patients, which would not stand the sequelae of massive immediate immune activation. Our finding that PBMCs from STS patients receiving poly-chemotherapy showed profound NK and T cell activation after stimulation with NKG2D-CD16 indicates that combinatorial therapy might be promising. Due to their high anti-tumor efficiency, NKG2D-CD3 BFP in turn might be effective as an adjuvant mono- or combination therapy. Similar to preliminary data on the combination of anti-PD1 and CAR-T cell therapy or bispecific antibodies, a combined immunotherapy could be conceivable for NKG2D-CD3 BFP treatment (44, 45). However, further data are needed to fully elucidate the bioavailability of our NKG2D BFP in the sarcoma TME in a clinical setting. For bispecific antibodies it is known that a PSMAxCD3 antibody applicated *in vivo* penetrates tumor tissue, specifically localizes at the tumor site and attracts immune effector cells (46).

In conclusion, NKG2D-CD16 and NKG2D-CD3 BFP showed powerful anti-sarcoma effects in a preclinical setting. Of note, our treatment approach was not restricted to a distinct STS entity, as different sarcoma cell lines including osteosarcoma, rhabdomyosarcoma and chondrosarcoma were sensitive to treatment with both types of BFP. Moreover, we observed that even if total account of lymphocytes in the peripheral blood is decreased, NK and T cell activation *in vitro* was not significantly affected in STS patients receiving intense treatment. Even if further work is needed to fully characterize the potential of our constructs, the data presented in this study highlight the potential of BFP targeting NKG2DL in STS.

## Data Availability Statement

The raw data supporting the conclusions of this article will be made available by the authors, without undue reservation.

## Ethics Statement

The study was approved by IRB (ethics committee of the Faculty of Medicine of the Eberhard Karls Universitaet Tuebingen and of the University Hospital Tuebingen) and was conducted in accordance with the Declaration of Helsinki; reference number 13/2007V and 612/2010BO2. Human material was collected after the patients/participants provided their written informed consent to participate in this study.

## Author Contributions

IH and ML designed and performed the experiments, analyzed and interpreted data, and wrote the manuscript. MS and JH collected patient samples and provided clinical data. EM, YZ, and KC designed and performed experiments. H-GK and GJ provided patient samples/reagents and contributed to the study design. HS contributed to the study design, critically revised the manuscript, and supervised the study. MM contributed to the study design, provided important advice, and critically revised the manuscript. CH collected clinical samples and provided patient data, contributed to the study design, wrote the manuscript, and supervised the study. All authors contributed to the article and approved the submitted version.

## Funding

This work was supported by ƒortüne junior grant (2478-0-0) as well as grants from DFG (SA1360/7–3), Germany’s Excellence Strategy (EXC 2180/1), Wilhelm Sander-Stiftung (2007.115.3), and Deutsche Krebshilfe (111828, 111134, 70112914). We acknowledge support by Deutsche Forschungsgemeinschaft and Open Access Publishing Fund of University of Tübingen.

## Conflict of Interest

The authors declare that the research was conducted in the absence of any commercial or financial relationships that could be construed as a potential conflict of interest.
