# Supplementary material for: Bispecific NKG2D-CD3 and NKG2D-CD16 Fusion Proteins as Novel Treatment Option in Advanced Soft Tissue Sarcomas
Source: Front Immunol. 2021 Apr 14;12:653081. doi: 10.3389/fimmu.2021.653081 (PMC8079770; doi:10.3389/fimmu.2021.653081)
Supplement: Supplementary file 1 [file DataSheet_1.docx]

Supplementary Material

# Supplementary Figures


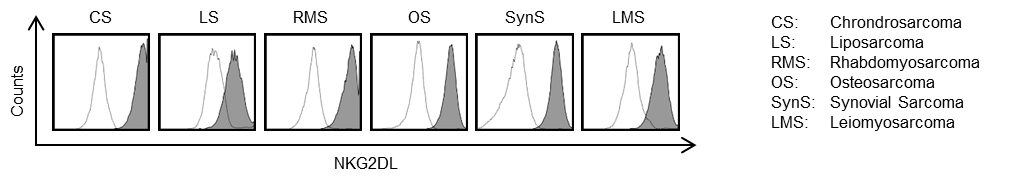


**Supplementary Figure 1** Surface expression of NKG2DL on the indicated patient-derived sarcoma cells was examined using an antibody cocktail containing antibodies against MICA, MICB, ULBP1-4 and ULBP2/5/6 by flow cytometry. mAb against the NKG2DL are shown as shaded peaks, corresponding isotype control is shown as open peaks.


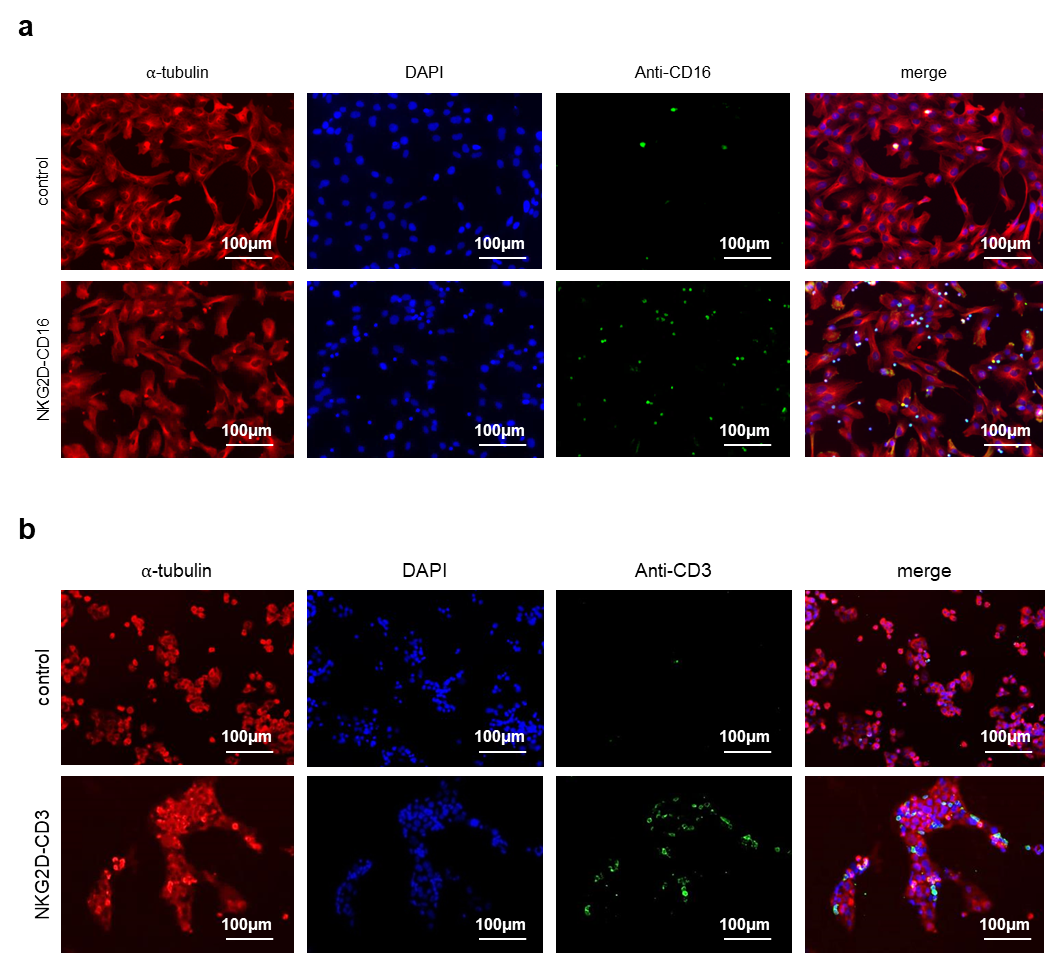


**Supplementary Figure 2** Sarcoma cell lines SW1353 and RD-ES were cultivated with PBMC of healthy donors (E:T 2.5:1) in the presence or absence of **a:** NKG2D-CD16 with SW1353 or **b:** NKG2D-CD3 (2.5 µg/mL) with RD-ES for 45 min. After fixation, cells were stained for α-tubulin (red; Alexa-633), DAPI (blue) and CD16 or CD3 (green; Alexa-488).


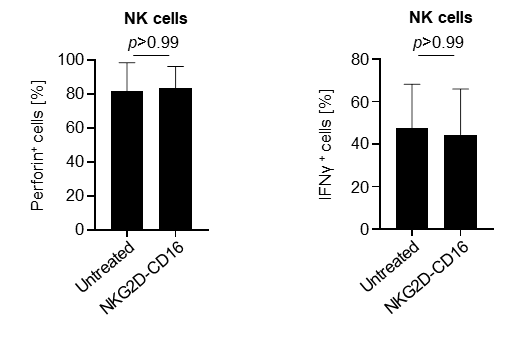


**Supplementary Figure 3** PBMC of healthy donors (n=6) were incubated with different sarcoma cell lines (RD-ES, SW1353, SaOs) and treated with the indicated constructs (2.5 µg/mL) for 24 h. Intracellular Perforin and IFNγ expression of NK cells was analyzed by flow cytometry.


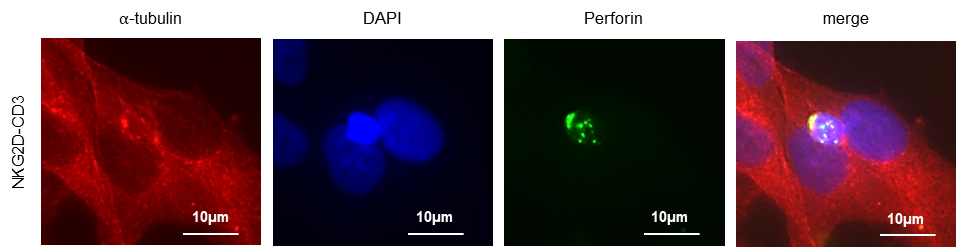


**Supplementary Figure 4** Sarcoma cells (SW1353) were cultivated with healthy PBMC (E:T 2.5:1) in the presence or absence of NKG2D-CD16/CD3 (2.5 µg/mL) for 1 h and 3 h, respectively. Cells were stained for α-Tubulin (red; Alexa-633), DAPI (blue) and the granular marker perforin (green; Alexa-488).


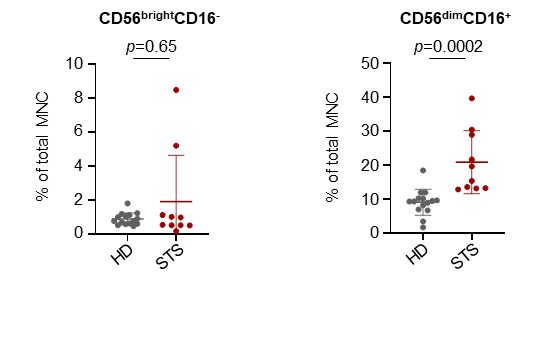


**Supplementary Figure 5** PBMC were collected from healthy donors (n=15) and patients with advanced STS (n=10). Indicated cell types were identified by counterstaining for CD3, CD56 and CD16.

**
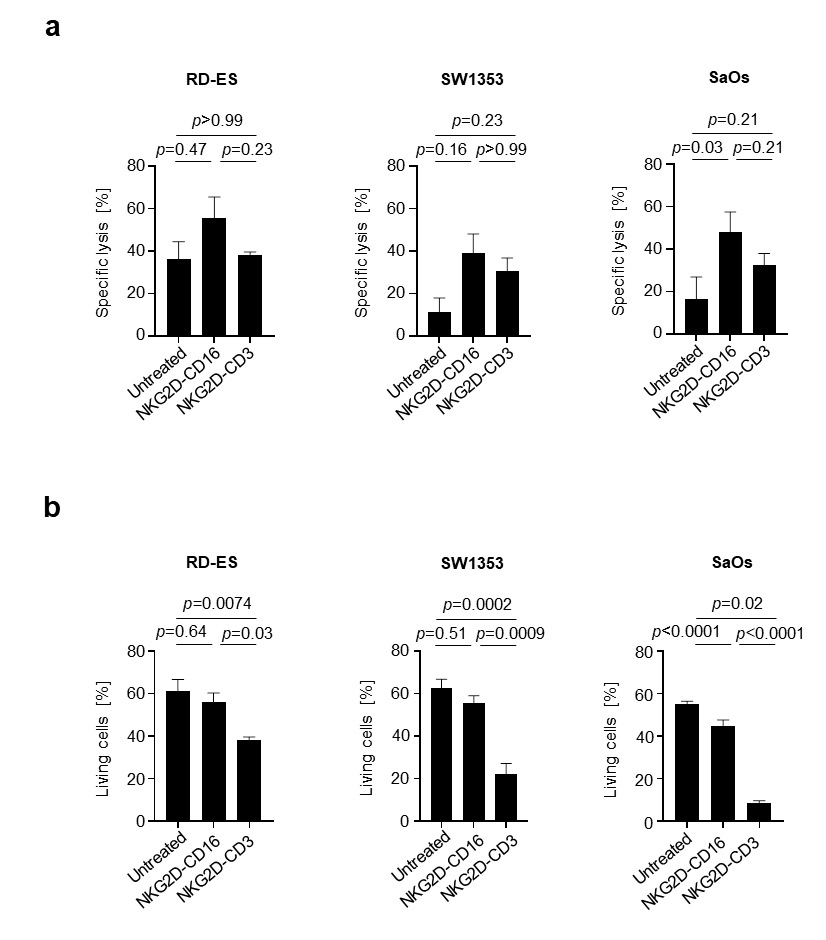
**

**Supplementary Figure 6** PBMC of healthy donors (n=4) were incubated with different sarcoma cell lines and treated with the indicated constructs (2.5 µg/mL). a: Lysis of sarcoma cell lines SaOS, RD-ES and SW1353 (n=3) was analyzed by 2 h Europium cytotoxicity assays. Exemplary data obtained with the indicated cell line and with different E:T ratios are shown. b: Lysis of sarcoma cell lines SaOS, RD-ES and SW1353 (n=3) was determined by flow cytometry based lysis assay (E:T 2.5:1) after 72 h using PBMC of healthy donors. Exemplary results with the indicated cell lines are shown.

# Supplementary Tables

**Supplemental Table 1** Soft-tissue sarcoma (STS) cell lines

| **Cell line** | **STS subtype** | **Karyotype** | **Known mutations** |
| --- | --- | --- | --- |
| SW982 | Synovial sarcoma (SS) | hyperdiploid | BRAF p.V600E Het. CDKN2A p.0? Hom. |
| SW872 | Liposarcoma (LS) | hypertriploid | BRAF p.V600E Het.  CDKN2A p.R80 Hom.  PTEN p.0? Hom.  TP53 p.I251N Hom. |
| RD-ES | Rhabdomyosarcoma (RMS) | unstable | NRAS p.Q61H Hom.  TP53 p.R248W Hom. |
| SW1353 | Chondrosarcoma (CS) | hyperdiploid | nd |
| SaOs | Osteosarcoma (OS) | hypotriploid | TP53 p.0? Hom.  Rb1 p.? Hom. |

**Supplemental Table 2** List of sarcoma samples used in this study

| **Patient sample** | **STS subtype** | **Gender** | **Age** | **Histopathological Grade (1-3)** |
| --- | --- | --- | --- | --- |
| LMS | Leiomyosarcoma (LMS) | F | 76 | 3 |
| OS | Osteosarcoma (OS) | M | 14 | 3 |
| RMS | Rhabdomyosarcoma (RMS) | M | 51 | 3 |
| LS | Liposarcoma (LS) | F | 58 | 1 |
| CS | Chrondrosarcoma (CS) | F | 62 | 3 |
| SynS | Synovial sarcoma (SynS) | M | 76 | 3 |

**Supplemental Table 3** Treatment regimens of sarcoma patients

| **Sarcoma patient** | **Treatment** |
| --- | --- |
| I | Anthracycline / platinum |
| II | Anthracycline / platinum |
| III | Anthracycline |
| VI | Vincaalcaloid / actinomycin |
| V | Vincaalcaloid / anthracycline / topoisomerase II inhibitor |
| VI | Anthracycline / platinum |
| VII | Anthracycline |
| VIII | Anthracycline / platinum |
| IX | Alkaloid (Trabectedin) |
| X | Alkylating agent / Purine analog (Dacarbazin) |
| XI | Vincaalcaloid / anthracycline / topoisomerase II inhibitor |
| XII | Anthracycline |
| XIII | Anthracycline / platinum |
| XIV | Anthracycline |
| XV | Alkaloid (Trabectedin) |
| XVI | Anthracycline |

**Supplemental Table 4** Primer sequences

| MICA | 5’-GGCATCTTCCCTTTTGCAC-3’ |
| --- | --- |
|  | 5’-GGACAGCACCGTGAGGTTAT-3’ |
| MICB | 5’-CTGAGAAGGTGGCGACGTA-3’ |
|  | 5’-CGAAGACTGTGGGGCTCA-3’ |
| ULBP1 | 5’-ACTGGGAACAAATGCTGGAT-3’ |
|  | 5’-GAGAAGGCTCCAGGGACTG-3’ |
| ULBP2 | 5’-CCGCTACCAAGATCCTTCTG-3’ |
|  | 5’-GGGATGACGGTGATGTCATAG-3’ |
| ULBP3 | 5’-TCCCTGGCATCTGAGAAGAG-3’ |
|  | 5’-CAGAAAGGCACAGTGGTGAGT-3’ |
| ULBP4 | 5’-AGCACTTGGGGAGAATTGAC-3’ |
|  | 5’-CTTGCAGAGTGGAAGGATCAC-3’ |
| GAPDH | 5’-AGCCACATCGCTCAGACAC-3’ |
|  | 5’-GCCCAATACGACCAAATCC-3’ |
